# Sustained Effectiveness and Safety of Therapeutic miR-10a/b in Alleviating Diabetes and Gastrointestinal Dysmotility without Inducing Cancer or Inflammation in Murine Liver and Colon

**DOI:** 10.3390/ijms25042266

**Published:** 2024-02-14

**Authors:** Rajan Singh, Se Eun Ha, Han Sung Park, Sushmita Debnath, Hayeong Cho, Gain Baek, Tae Yang Yu, Seungil Ro

**Affiliations:** 1 Department of Physiology and Cell Biology, School of Medicine, University of Nevada, Reno, NV 89557, USA; rajans@med.unr.edu (R.S.); seeunh@med.unr.edu (S.E.H.); hansungp@unr.edu (H.S.P.); sdebnath@unr.edu (S.D.); hayeongc@unr.edu (H.C.); gbaek@unr.edu (G.B.); taeyangy@unr.edu (T.Y.Y.); 2 RosVivo Therapeutics, Applied Research Facility, 1664 N. Virginia St., Reno, NV 89557, USA

**Keywords:** miR-10a-5p, miR-10b-5p, diabetes, gastrointestinal dysmotility, cancer, inflammation

## Abstract

microRNAs (miRNAs) are key regulators of both physiological and pathophysiological mechanisms in diabetes and gastrointestinal (GI) dysmotility. Our previous studies have demonstrated the therapeutic potential of miR-10a-5p mimic and miR-10b-5p mimic (miR-10a/b mimics) in rescuing diabetes and GI dysmotility in murine models of diabetes. In this study, we elucidated the safety profile of a long-term treatment with miR-10a/b mimics in diabetic mice. Male C57BL/6 mice were fed a high-fat, high-sucrose diet (HFHSD) to induce diabetes and treated by five subcutaneous injections of miR-10a/b mimics for a 5 month period. We examined the long-term effects of the miRNA mimics on diabetes and GI dysmotility, including an assessment of potential risks for cancer and inflammation in the liver and colon using biomarkers. HFHSD-induced diabetic mice subcutaneously injected with miR-10a/b mimics on a monthly basis for 5 consecutive months exhibited a marked reduction in fasting blood glucose levels with restoration of insulin and significant weight loss, improved glucose and insulin intolerance, and restored GI transit time. In addition, the miR-10a/b mimic-treated diabetic mice showed no indication of risk for cancer development or inflammation induction in the liver, colon, and blood for 5 months post-injections. This longitudinal study demonstrates that miR-10a/b mimics, when subcutaneously administered in diabetic mice, effectively alleviate diabetes and GI dysmotility for 5 months with no discernible risk for cancer or inflammation in the liver and colon. The sustained efficacy and favorable safety profiles position miR-10a/b mimics as promising candidates in miRNA-based therapeutics for diabetes and GI dysmotility.

## 1. Introduction

Diabetes, a global health challenge, is increasingly recognized for its association with gastrointestinal (GI) complications, notably GI dysmotility [1,2,3]. This linkage underscores an intricate interplay between metabolic dysfunction and gut health [2]. Current therapeutic strategies largely focus on symptomatic relief, often falling short in addressing the underlying cellular dysfunctions that contribute to both diabetes and GI dysmotility [4,5]. Thus, there is an imperative need for treatments that target the root causes of these diseases.

Recent studies in cellular biology have illuminated the critical importance of cell types in regulating both glucose homeostasis and GI function [5,6]. Notably, pancreatic β cells and gastrointestinal pacemaking cells, known as interstitial cells of Cajal (ICC), play pivotal roles in these intricate processes [5,7,8]. Dysfunctions in these cells are key contributors in the development of diabetes and GI dysmotility, stemming from disturbances in regulatory RNA mechanisms [9,10]. microRNAs (miRNAs) emerge as pivotal regulators governing the cellular functions of pancreatic β cells and ICC [11,12,13,14,15].

Our previous research has shed light on the essential role of miR-10a-5p and miR-10b-5p (miR-10a/b-5p) in pancreatic β cells and ICC [16,17]. We discovered that miR-10a/b-5p are the most highly expressed in receptor tyrosine kinase protein KIT^+^ ICC in healthy mice and drastically depleted in ICC in diabetic *ob*/*ob* mice [16]. We further found that loss of miR-10b-5p in KIT^+^ β cells and ICC in *Kit-mir-10b* KO mice led to diabetes and GI dysmotility, revealing a novel miR-10b-KLF11-KIT pathway that regulates glucose homeostasis and GI motility [16]. In addition, we found that miR-10a-5p mimic and miR-10b-5p mimic (miR-10a/b mimics) intervention effectively reversed these pathological phenotypes in the multiple models of diabetic mice by restoration of β cells and ICC, highlighting the potential utilization of miR-10a/b mimics in therapeutic applications [16,17].

However, there is a safety concern in the potential therapeutic application of miR-10a/b mimics. Overexpression of miR-10a/b-5p is associated with various types of cancer, raising concerns about its potential oncogenic effect [18,19,20,21]. In this study, we conducted a comprehensive assessment of the efficacy and safety profiles of miR-10a/b mimics, monitoring the long-term effect on diabetes and GI dysmotility and the risk for cancer and inflammation in the liver and colon using biomarkers in diabetic mice injected with miR-10a/b mimics monthly for a 5 month period.

## 2. Results

### 2.1. Long-Term Rescue of Diabetes by miR-10a/b Mimics without Inducing Overexpression of miR-10a/b-5p

**Figure 1:**
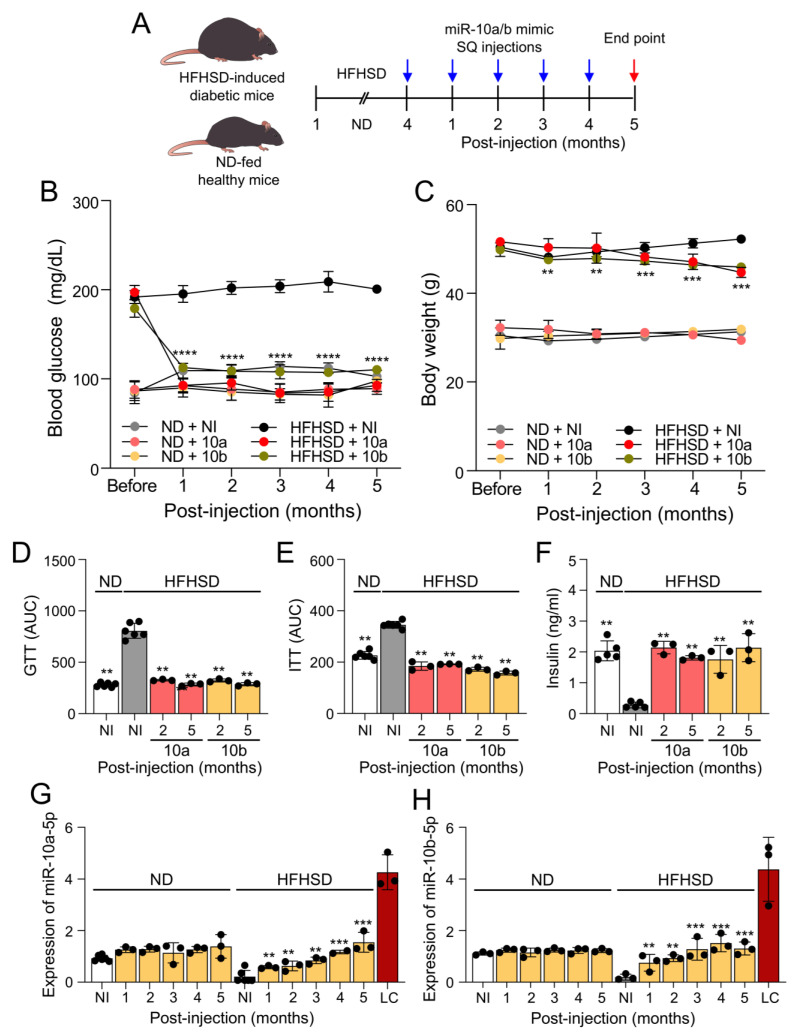
Long-term treatment with miR-10a/b mimics rescues diabetes in high-fat, high-sucrose diet-induced diabetic mice with no observed overexpression of miR-10a/b-5p. (**A**) Outline of the study. Male mice were fed a high-fat, high-sucrose diet (HFHSD) or a normal diet (ND) for 4 months and subcutaneously (SQ) injected with miR-10a-5p mimic or miR-10b-5p mimic (miR-10a/b mimics) each month for a 5 month period. (**B**) Fasting blood glucose comparison in HFHSD-induced diabetic mice and ND-fed healthy mice injected with miR-10a/b mimics or given no injection (NI) over a 5 month period post-injection (PI). (**C**) Body weight comparison in HFHSD-induced diabetic and ND-fed healthy mice injected with miR-10a/b mimics or given NI. (**D**,**E**) Glucose and insulin tolerance test (GTT and ITT) plots of the area under the curve (AUC) comparison in HFHSD-induced diabetic and ND-fed healthy mice injected with miR-10a/b mimics or given NI. (**F**) Comparison of 6 h fasting insulin levels in plasma from HFHSD-induced diabetic and ND-fed healthy mice injected with miR-10a/b mimics or given NI. (**G**,**H**) Levels of miR-10a-5p and miR-10b-5p in whole blood samples from HFHSD-induced diabetic and ND-fed healthy mice injected with miR-10a/b mimics or given NI and liver cancer (LC). n = 3–6 per condition for each experiment. Error bar indicates mean ± SEM, two-way analysis of variance (ANOVA). ** *p* < 0.01; *** *p* < 0.001; **** *p* < 0.0001.

To examine the long-term effect of miR-10a/b mimics on diabetes, we used mice that were fed a high-fat, high-sucrose diet (HFHSD) for 4 months to induce diabetes along with healthy mice that were fed a normal diet (ND). HFHSD-induced diabetic and ND-fed healthy mice were subcutaneously injected with miR-10a-5p mimic or miR-10b-5p mimic monthly for 5 months (Figure 1A). HFHSD-fed mice became hyperglycemic and obese compared to those fed with a ND (Figure 1B,C). Notably, the HFHSD-induced diabetic mice after miR-10a/b mimic injections showed a marked reduction in fasting blood glucose levels with significant weight loss compared to untreated diabetic mice (Figure 1B,C) and diabetic mice injected with a negative control scramble RNA (Supplementary Figure S1). Blood glucose levels in HFHSD-induced diabetic mice after miR-10a/b mimic injections were dramatically reduced to normal levels at 1 month post-injection (PI), which were maintained up to 5 months PI (Figure 1B). Healthy mice injected with miR-10a/b mimics for five consecutive months also maintained normal blood glucose levels but did not develop hypoglycemia (Figure 1B). HFHSD-induced diabetic mice after miR-10a/b mimic injections gradually reduced body weight over 5 months while ND-fed healthy mice after miR-10a/b mimic injections maintained normal body weight (Figure 1C).

HFHSD-induced diabetic mice exhibited impaired glucose and insulin tolerance, both of which were significantly improved after miR-10a/b mimic injections at 2 and 5 months (Figure 1D,E). Insulin was drastically attenuated in HFHSD-induced diabetic mice but was restored to normal levels after miR-10a/b mimic injections at 2 and 5 months (Figure 1F). miR-10a/b-5p levels were substantially reduced in the blood of HFHSD-induced diabetic mice, but were increased to normal levels after miR-10a/b mimic injections as seen in ND-fed healthy mice (Figure 1G,H). However, miR-10a/b mimic injections in ND-fed healthy mice did not result in overexpression of miR-10a/b-5p levels. Monthly miR-10a/b mimic injections in both HFHSD-induced diabetic mice and ND-fed healthy mice did not result in miR-10a/b-5p levels being elevated to oncogenic levels of liver cancer. These data demonstrate that subcutaneous miR-10a/b mimic injections in HFHSD-induced diabetic mice effectively rescue the diabetic phenotypes for 5 months without inducing excessive miR-10a/b-5p levels.

### 2.2. Long-Term Rescue of GI Dysmotility by miR-10a/b Mimics in HFHSD-Induced Diabetic Mice without Indication of Risk for Colon Cancer

**Figure 2:**
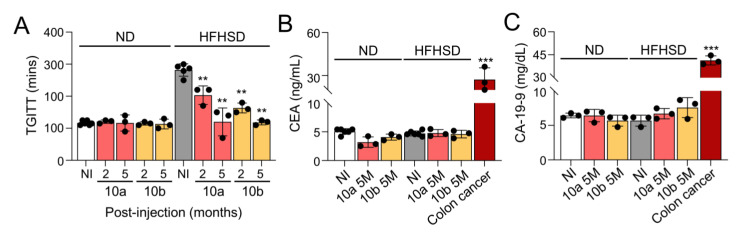
Long-term treatment with miR-10a/b mimics rescues gastrointestinal dysmotility in HFHSD-induced diabetic mice without indication of increased risk for colon cancer. (**A**) Comparison of total gastrointestinal transit time (TGITT) in HFHSD-induced diabetic and ND-fed healthy mice after miR-10a/b mimic injections or no injection (NI) at 2 and 5 months (2M and 5M). (**B**) Comparison of carcinoembryonic antigen (CEA) levels in the blood samples from HFHSD-induced diabetic and ND-fed healthy mice after miR-10a/b mimic injections or NI at 5 months and in human colorectal adenocarcinoma Caco-2 cells. (**C**) Comparison of carbohydrate antigen 19-9 (CA-19-9) levels in the colon tissue and in Caco-2 cells. n = 3–6 per condition for each experiment. Error bar indicates mean ± SEM, two-way ANOVA. ** *p* < 0.01; *** *p* < 0.001.

To examine the long-term effect of miR-10a/b mimics on GI dysmotility, we performed total GI transit time tests in HFHSD-induced diabetic mice and ND-fed healthy mice. Total GI transit time was noticeably delayed in HFHSD-induced diabetic mice, but significantly decreased after miR-10a/b mimic injections at 2 months and further decreased to normal GI transit times at 5 months as seen in ND-fed healthy mice (Figure 2A). Next, we performed a colorectal cancer screening test in HFHSD-induced diabetic mice and ND-fed healthy mice with five consecutive miR-10a/b mimic injections at 5 months PI using the well-known tumor markers CEA and CA-19-9 [22], which are widely used in the diagnosis of colon cancer. Both CEA and CA-19-9 were notably elevated in human colorectal adenocarcinoma Caco-2 cells, but not in the blood and colonic tissue from HFHSD-induced diabetic mice and ND-fed healthy mice after five consecutive miR-10a/b mimic injections at 5 months (Figure 2B,C). Taken together, the data indicate that the long-term treatment with miR-10a/b mimics can rescue delayed total GI transit in diabetic mice without indication of increased risk for colon cancer.

### 2.3. Long-Term Treatment with miR-10a/b Mimics in HFHSD-Induced Diabetic Mice Indicates No Risk for Liver Cancer

**Figure 3:**
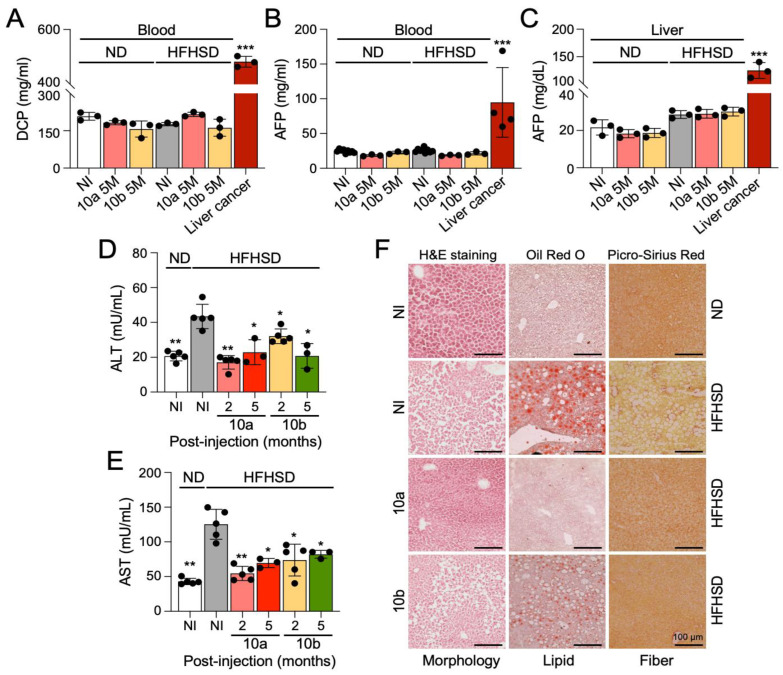
Long-term treatment with miR-10a/b mimics in HFHSD-induced diabetic mice indicates no increased risk for liver cancer. (**A**) Comparison of des-gamma-carboxy prothrombin (DCP) levels in blood samples from HFHSD-induced diabetic and ND-fed healthy mice after miR-10a/b mimic injections or no injection (NI) at 5 months and mice with liver cancer. (**B**) Comparison of alpha-fetoprotein (AFP) levels in blood samples. (**C**) Comparison of alpha-fetoprotein (AFP) levels in liver tissue. (**D**,**E**) Comparison of liver function (ALT and AST levels) in blood samples after miR-10a/b mimic injections or NI at 2 and 5 months. (**F**) Liver cryostat sections of H&E, Oil Red O, and Picro-Sirius Red for comparison of morphology, lipid droplets, and fiber content in the liver tissue after miR-10a/b mimic injections or NI at 5 months. Scale bars are 100 μm. n = 3 per condition for each experiment. Error bar indicates mean ± SEM, two-way ANOVA. * *p* < 0.05; ** *p* < 0.01; *** *p* < 0.001.

To examine an oncogenic effect of miR-10a/b mimics on liver cancer, we performed a liver cancer screening test in HFHSD-induced diabetic mice and ND-fed healthy mice after five consecutive miR-10a/b mimic injections by using the tumor markers, des-gamma-carboxy prothrombin (DCP) and alpha-fetoprotein (AFP), which are widely used to detect hepatocellular carcinoma [23]. Both DCP and AFP levels were elevated in the blood of mice with liver cancer, but not in HFHSD-induced diabetic mice and ND-fed healthy mice after five consecutive miR-10a/b mimic injections at 5 months (Figure 3A,B). Similarly, AFP levels were detected in the liver cancer tissue at high levels but were detected at lower levels in the liver of miR-10a/b mimic-injected diabetic mice and healthy mice (Figure 3C). Next, we performed the liver function test using alanine transaminase (ALT) and aspartate aminotransferase (AST) markers, which are routinely used to detect liver dysfunction and damage [24], in HFHSD-induced diabetic mice and ND-fed healthy mice after five miR-10a/b mimic injections. Both ALT and AST were significantly reduced in the blood from HFHSD-induced diabetic mice after miR-10a/b mimic injections at 2 and 5 months (Figure 3D,E). Furthermore, Oil Red O and Picro-Sirius Red stainings of liver cryostat sections revealed an excessive accumulation of lipids and fibers in the liver tissue of HFHSD-induced diabetic mice, but accumulation was noticeably reduced in the liver tissue of miR-10a mimic-injected diabetic mice at 5 months PI (Figure 3F). miR-10b mimic injected diabetic mice also showed a decrease in lipids and fibers, although the reduction in lipids was less notable compared to use of the miR-10a mimic. These findings suggest that the long-term treatment with miR-10a/b mimics can reverse fatty and fibrous liver or liver damages in diabetic mice without indication of increased risk for liver cancer.

### 2.4. Long-Term Treatment with miR-10a/b Mimics in HFHSD-Induced Diabetic Mice Indicates No Risk for Inflammation in Colon and Liver

**Figure 4:**
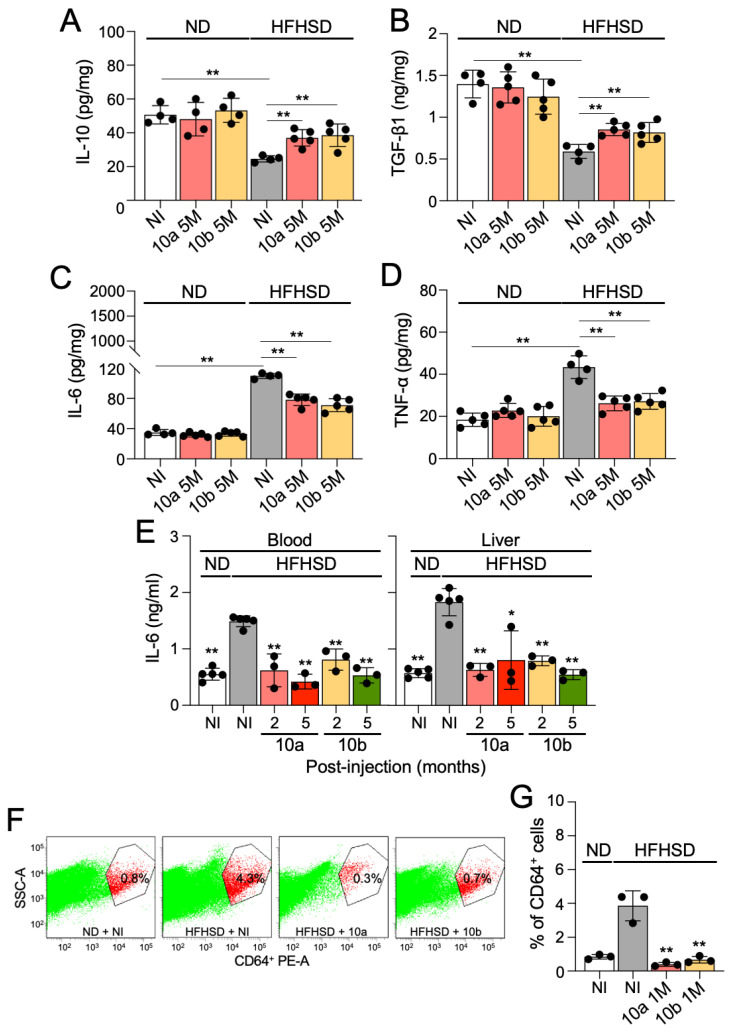
Long-term treatment with miR-10a/b mimics in HFHSD-induced diabetic mice indicates no risk for inflammation in the colon and liver. (**A**) Comparison of interleukin 10 (IL-10) levels in colon tissue from HFHSD-induced diabetic and ND-fed healthy mice after miR-10a/b mimic injections or no injection (NI) at 5 months. (**B**) Comparison of transforming growth factor beta 1 (TGF-β1) levels in colon tissue. (**C**) Comparison of interleukin (IL-6) levels in the colon tissue. (**D**) Comparison of tumor necrosis factor alpha (TNF-α) levels in the colon tissue. (**E**) Comparison of IL-6 levels in the blood and liver tissue from HFHSD-induced diabetic and ND-fed healthy mice after miR-10a/b mimic injections or NI at 2 and 5 months. (**F**,**G**) Pro-inflammatory macrophage (CD64^+^) population identified by flow cytometry in the liver of HFHSD-induced diabetic and ND-fed healthy mice injected after miR-10a/b mimic injections or NI at 1 month. n = 3–5 per condition for each experiment. Error bar indicates mean ± SEM, two-way ANOVA. * *p* < 0.05; ** *p* < 0.01.

To provide a comprehensive assessment of the safety profile of mice treated with miR-10a/b mimics, we further assessed inflammation in the colon and liver by measuring anti-/pro-inflammatory cytokines in miR-10a/b mimic-injected diabetic mice. Both anti-inflammatory cytokines, interleukin 10 (IL-10) and transforming growth factor-beta1 (TGF-β1), were noticeably decreased in the colon of HFHSD-induced diabetic mice compared to ND-fed healthy mice, but were restored to normal levels in miR-10a/b mimic-injected diabetic mice 5 months PI (Figure 4A,B). In contrast, both pro-inflammatory cytokines, IL-6 and tumor necrosis factor-alpha (TNF-α), were substantially increased in the colon of HFHSD-induced diabetic mice compared to ND-fed healthy mice, but were significantly decreased in the colon of miR-10a/b mimic-injected diabetic mice 5 months PI (Figure 4C,D). In addition, IL-6 was significantly reduced in the blood and liver of diabetic mice after miR-10a/b mimic injections at 2 and 5 months PI (Figure 4E). We further examined the number of inflammatory macrophages in the liver of miR-10a/b mimic-injected diabetic mice and found that the population of pro-inflammatory macrophages (CD64^+^) was noticeably increased in the liver of HFHSD-induced diabetic mice compared to ND-fed healthy mice, but was significantly decreased in the liver of miR-10a/b mimic-injected diabetic mice 1 month PI (Figure 4F,G). These inflammation marker tests in the colon, liver, and blood confirm that the long-term treatment with miR-10a/b mimics in HFHSD-induced diabetic mice indicates no risk for inflammation in the colon and liver. 

## 3. Discussion

This study has explored the therapeutic potential and safety of miR-10a/b mimics in treating diabetes and GI dysmotility. Our results indicate that the long-term treatment of HFHSD-induced diabetic mice subcutaneously injected with miR-10a/b mimics can effectively rescue both diabetes and GI dysmotility without indication for increased risk of cancer development and inflammation induction. These efficacy and safety data are consistent with our previous studies [16,17] that highlighted the pivotal role of miR-10a/b-5p in regulating glucose homeostasis and GI motility as well as the promising efficacy of miR-10a/b mimics in reversing diabetes and GI dysmotility without induction of cancer. 

In our previous study, we tested the safety of miR-10b-5p mimic at two therapeutic dosages of 500 ng/g injected in healthy mice and observed no indication of cancer development in the mice over an extended period of one year [16]. We further demonstrated that a single injection of the miR-10b-5p mimic into HFHSD-fed diabetic mice restored miR-10b-5p levels in blood to approximately 40–60% of normal, healthy levels but not to the excessively elevated levels reported in cancers [18,19] and in the liver cancer that we tested in the study [16]. In addition, the serum stability of miR-10b mimic in vitro and in vivo in rodents is transient with approximately 50% of the miRNAs being lost within one hour (unpublished data). Thus, it is unlikely that miR-10b-5p injections will lead to the risk of liver and colon cancer when used to treat diabetes and GI dysmotility caused by substantially reduced miR-10b-5p expression, as observed in HFHSD-induced diabetic mice.

An essential aspect of the current study was assessing the extended safety profile of a long-term miR-10a/b mimic treatment for diabetic mice, particularly the risk pertaining to cancer and inflammation. Our longitudinal study, encompassing blood- and tissue-based cancer biomarker screening, showed no indication of risk for cancer development and inflammation induction in the liver, colon, or blood. Notably, although overexpression of miR-10a/b-5p has been linked to cancer in various contexts [18,19,20], our study indicates that the five monthly doses of 500 ng/g of miR-10a/b mimics subcutaneously injected in diabetic mice are safe as they did not reach the excessive oncogenic levels of miR-10a/b-5p.

miR-10a/b-5p are bifunctional in cancer as they function as both tumor suppressors and oncogenes, inhibiting or promoting cancer development and progression depending on the cellular contexts and the genes they target [25,26]. Overexpression of miR-10a/b-5p is associated with various types of cancers, contributing to enhanced cell proliferation and migration [18,19,20]. However, it is also well-documented that miR-10a/b-5p are downregulated in certain cancers and inhibit tumorigenicity [27,28,29,30]. miR-10a/b-5p have both oncogenic and tumor-suppressive roles in gastric cancer [26], colorectal cancer [31], breast cancer [32], and gynecological malignancies [25]. The dual function of miR-10a/b-5p is dependent on their target genes. For example, miR-10a/b-5p are encoded within the *Hox* clusters of developmental regulators and regulate the translation of *Hox* transcripts [33]. *HOX* genes have both tumor suppressor and pro-oncogenic activities [34]. miR-10a/b-5p also target and suppress the protein translation of Krüppel-like factor 11 (KLF11), which induces apoptosis or cell cycle arrest [16,35]. KLF11 also has a dual function in cell growth and cancer as a tumor suppressor and a tumor promoter [35]. In our previous study, we demonstrated a novel mechanistic pathway, miR-10a/b-5p-KLF11-KIT, in the regulation of glucose homeostasis and GI motility. We found that miR-10a/b-5p induces the growth of ICC and pancreatic β cells by targeting KLF11 (a causative gene for maturity-onset of diabetes of the young), which negatively regulates expression of *KIT* and insulin (*INS*) genes. We previously confirmed that miR-10a/b-5p mimics directly target KLF11 in ICC and pancreatic β cells, increasing KIT and INS protein [16]. KIT functions as a proto-oncogene via its kinase activity, as well as a tumor suppressor via its receptor activity [36]. KIT is required for the normal growth and differentiation of ICC, but excessive KIT triggers gastrointestinal stromal tumor [37]. 

Our current study observed a restoration of colonic and liver anti-inflammatory cytokines in mice treated with miR-10a/b mimics, suggesting an anti-inflammatory role of these miRNAs. This observation aligns with previous studies that have confirmed the anti-inflammatory function of miR-10a/b-5p in both murine and human gut inflammatory conditions [38,39,40,41,42,43]. Specifically, murine studies have shown that a deficiency of miR-10a/b-5p can exacerbate dextran sodium sulfate-induced inflammatory responses by impairing intestinal barrier function [38]. Additionally, studies found downregulation of miR-10a/b-5p in patients with inflammatory bowel disease, particularly in patients with colitis [41,43]. Furthermore, previous studies have demonstrated that miR-10a/b-5p might regulate macrophage functions, facilitating a transition in their phenotypes from pro-inflammatory to anti-inflammatory [44,45]. The ability of miR-10a/b-5p to alleviate these inflammatory phenotypes and reduce inflammation holds significant promise for clinical applications, especially in treating patients with both diabetes and GI dysmotility.

The role of miR-10a/b extends beyond the phenotypic regulation of pancreatic β cells and ICC. miR-10a/b are also essential for intestinal barrier function, which is crucial for GI and metabolic homeostasis [2]. Our previous study revealed that global *mir-10b* KO mice displayed impaired intestinal barrier function, characterized by a disorganized epithelial barrier and increased gut permeability [17]. Intervention with a miR-10b mimic in these mice rescued the hyperglycemic GI dysmotility and leaky gut phenotype through remodeling epithelial cells, thereby maintaining GI homeostasis and metabolic health [17]. Our findings demonstrated how miR-10a/b mimic treatment may beneficially restore GI pathophysiology under diabetic conditions.

The implications of our study are far-reaching, highlighting the therapeutic potential of miR-10a/b mimics for disorders associated with reductions in or deficiency of these miRNAs. By targeting the underlying causes (dysfunction or reduction in pancreatic β cells and ICC) of diabetes and GI dysmotility, miR-10a/b mimics offer a more comprehensive approach compared to current treatments that focus largely on symptomatic treatment and temporary relief. Furthermore, we have explored the therapeutic potential of miR-10a/b mimics delivered via a subcutaneous injection in mice, which aligns with the translational approach to diabetes treatment, as current diabetic medications, such as GLP1 receptor agonists (liraglutide and semaglutide), also use subcutaneous injections [46,47]. Our findings, demonstrating the efficacy of subcutaneously delivered miR-10a/b mimics in lowering blood glucose levels and body weight, mirror the results from our previous study using miR-10a/b mimics that were administered intraperitoneally. Furthermore, in our previous study, we compared the efficacy of miR-10b mimic against diabetes in HFHSD-induced diabetic mice with antidiabetic medications [insulin, liraglutide, DPP-4 inhibitor (sitagliptin), and metformin] [16]. HFHSD-induced diabetic mice with two miR-10b-5p mimic injections significantly reduced body weight and effectively rescued the hyperglycemic condition. Antidiabetic medications also effectively lowered blood glucose, but the efficacy and duration were lower and shorter than that of miR-10b-5p mimic. Furthermore, GTT and ITT were improved in miR-10b-5p mimic-injected mice for up to 8 weeks after two injections, while the antidiabetic medications temporally improved GTT and ITT within 4 weeks during treatment. Our drug comparison study with the miR-10b-5p mimic depicted a compelling long-term efficacy in reversing diabetic phenotypes when compared to commonly used antidiabetic medications. This study paves the way for further research, especially in optimizing miR-10a/b mimic dosages and frequencies and investigating their long-term effects on various metabolic conditions including obesity and fatty liver disease. Future studies could also explore the applicability of these potential miR-10a/b mimics in other inflammatory diseases where they may alleviate the disease conditions.

While our findings are promising, it is important to acknowledge the limitations of our study. The research was conducted in a diet-induced murine model, and, thus, the translational applicability to human patients requires further investigations. Moreover, investigations are warranted to elucidate the therapeutic applications of miR-10a/b in metabolic disorders beyond diabetes and GI dysmotility, such as obesity, fatty liver disease, and diabetic nephropathy. Additionally, although we observed no increased risk for the development of cancer or inflammation in the liver and colon of HFHSD-induced diabetic mice after multiple injections of miR-10a/b mimics, additional studies in various cancer animal models are essential to fully evaluate the oncogenic risk of miR-10a/b. 

In conclusion, our study provides compelling evidence supporting the therapeutic potential of miR-10a/b mimics in addressing both diabetes and GI dysmotility. With their favorable safety profile and sustained efficacy in the long-term, miR-10a/b mimics emerge as promising candidates for RNA-based therapeutic interventions targeting diabetes and GI dysmotility.

## 4. Materials and Methods

### 4.1. Mice

*C57BL/6* male mice were obtained from Jackson Laboratory. The Institutional Animal Care and Use Committee at the University of Nevada, Reno (UNR) approved all experimental procedures. The colony of mice included in this study was housed in a centralized animal facility at the UNR Animal Resources. UNR is fully accredited by the American Association for Accreditation of Laboratory Animal Care International. Mice were air-freighted to UNR, where they were housed in the transgenic facility at the UNR School of Medicine. All mice were housed under pathogen-free conditions on a 12 h light/dark cycle with food and water ad libitum. Mice were euthanized by inhaling CO_2_, followed by cervical dislocation. A ventral midline incision was made, and the whole GI tract was carefully excised. These procedures were in accordance with National Institutes of Health guidelines for the care and use of laboratory animals.

### 4.2. Diet 

Mice were fed either a high-fat, high-sucrose diet (a representative western diet) (ENVIGO, Indianapolis, IN, USA) containing 14.7% kcal protein, 44.6% kcal fat, and 40.7% kcal carbohydrates or a purified Teklad normal diet (ENVIGO, Indianapolis, IN, USA) containing 20.5% kcal protein, 10.5% kcal fat, and 69.1% kcal carbohydrates for 5 months. 

### 4.3. miR-10a/b Mimic Intervention

A total of 500 ng/g of the miR-10a-5p mimic, miR-10b-5p mimic, or a negative control scramble RNA (Thermo Fisher Scientific, Waltham, MA, USA) was delivered into mice by subcutaneous (SQ) injection. In vivo-jetPEI (Polyplus-transfection) was used as the delivery agent. In vivo-jetPEI/miRNA complexes were prepared according to the manufacturer’s protocol, as previously described [16]. SQ injections were performed on mice using complexes equilibrated at room temperature.

### 4.4. Metabolic Procedure

Body weight and 6 h fasting blood glucose were measured and monitored monthly. A glucose tolerance test (GTT) was performed by measuring the 6 h fasting blood glucose levels as well as blood glucose levels at 15, 30, 60, 90, and 120 min post-glucose injection (2 g/kg body weight) [16]. An insulin tolerance test (ITT) was performed by measuring the 6 h fasting blood glucose levels as well as blood glucose levels at 30, 60, 90, and 120 min post-insulin glargine (Lantus, Bridgewater, NJ. USA) (0.75 IU/kg) injection [16]. Area under the curve analysis for both GTT and ITT was performed using GraphPad Prism 9 software.

### 4.5. Total Gastrointestinal Transit Time Test

The total gastrointestinal transit time (TGITT) test was performed on mice fasted overnight. Mice were orally gavaged with 0.1 mL of a semiliquid solution containing 5% Evans Blue in 0.9% NaCl and 0.5% methylcellulose. Mice were then monitored every 10 min until a fecal pellet containing the Evans Blue solution was expelled. TGITT was calculated as the time between the intragastric gavage of the dye and the visualization of the first blue fecal pellet [16].

### 4.6. Reverse Transcription Quantitative Polymerase Chain Reaction

Total RNAs were isolated from blood samples using the mirVana miRNA Isolation Kit (Ambion, Austin, TX, USA) as previously described [16]. RNA quality and quantity were evaluated using a Nanodrop 2000 Spectrophotometer (Thermo Fisher Scientific, Waltham, MA, USA), then total RNAs were reverse transcribed into complementary DNA (cDNA) using the TaqMan™ MicroRNA Reverse Transcription Kit (Thermo Fisher Scientific, Waltham, MA, USA). TaqMan Advanced MicroRNA Assay probes, namely, mmu-miR-10a-5p, mmu-miR-10b-5p, and mmu-U6 were used. A standard qPCR protocol was followed on qTOWER3 84 (Analytik Jena, Jena, Thuringia, Germany). The comparative cycle threshold method was used to compare relative transcription levels. The transcription level of each miRNA was estimated as the relative fold-change over the control U6 genes. All samples were run in triplicate for each assay.

### 4.7. Enzyme-Linked Immunosorbent Assay

Levels of des-gamma-carboxy prothrombin (DCP), alpha-fetoprotein (AFP), alanine transaminase (ALT), aspartate aminotransferase (AST), carcinoembryonic antigen (CEA), carbohydrate antigen 19-9 (CA-19-9), IL-10, TGF-β1, IL-6, TNF-α, and insulin were measured in blood or tissue from mice and in human colorectal adenocarcinoma Caco-2 cells (HTB-37™, ATCC, Manassas, VA, USA). Blood was collected by penetrating the retro-orbital sinus of mice. Blood was stored in a vial containing EDTA to prevent blood clotting and spun down at 15,000 rpm for 15 min at 4 °C in order to collect plasma. Collected plasma was stored at −80 °C. Liver and colonic tissues were homogenized in a radioimmunoprecipitation assay (RIPA) buffer using a Bullet Blender. The homogenate was then centrifuged at 12,000 rpm for 5 min at 4 °C. The supernatant was then collected and stored at −80 °C. A Bradford assay was then performed to measure the protein concentration of each sample. Enzyme-linked immunosorbent assays (ELISAs) were performed on plasma and/or tissue samples using ELISA kits for DCP (MyBioSource, San Diego, CA, USA), AFP, ALT, AST (Abcam, Cambridge, UK), CEA (MyBioSource, San Diego, CA, USA), CA-19-9 (Antibodies, Pottstown, PA, USA), IL-10, TGF-β, IL-6, TNF-α (Proteintech, Rosemont, IL, USA), and insulin (Crystal Chem, Elk Grove Village, IL, USA) according to the manufacturer’s protocol.

### 4.8. Immunohistochemical and Confocal Microscopy Analysis

Liver tissue was analyzed through cryostat-sectioned staining and confocal microscopy. Liver tissue was dissected in Krebs buffer (125.35 mmol/L NaCl, 5.9 mmol/L KCl, 1.2 mmol/L NaHPO_4_, 15.5 mmol/L NaHCO_3_, 1.2 mmol/L MgCl_2_, 11.5 mmol/L D-glucose, and 2.5 mmol/L CaCl_2_). Liver tissue was fixed in 4% paraformaldehyde at 4 °C for 20 min, followed by overnight incubation in 1 X Tris-buffered saline (TBS) at 4 °C. Dehydration was performed in 20% sucrose in TBS at 4 °C. Tissue was trimmed and placed in 1:1 optimum cutting temperature (OCT)/20% sucrose in TBS and frozen by liquid nitrogen. Then, 8 mm-thickness cryostat sections were prepared on slides and used for immunohistochemistry. Cryostat sections were blocked with 0.5% Triton X-114, 4% skim milk in TBS for 1h at room temperature, rinsed with TBS twice for 10 min each, and then incubated with anti-alpha fetoprotein (rabbit, 1:100, Proteintech, Rosemont, IL, USA) for 48 h on a rocker at 4 °C. The slides were rinsed with TBS twice for 10 min each and then incubated with 594-anti-rabbit (Jackson ImmunoResearch, West Grove, PA, USA) for 2 h at room temperature. The slides were rinsed with 1× TBS three times for 10 min each, dried, and mounted (mounting medium with 4′,6-diamidino-2-phenylindole (DAPI). An Olympus Fluoview FV1000 confocal laser scanning microscope (Olympus, Shinjuku, Tokyo, Japan) was used for all imaging analyses.

### 4.9. Hematoxylin and Eosin, Oil Red O, and Picro-Sirius Red Staining

Liver cryostat sections were stained with a hematoxylin and eosin (H&E) solution (Abcam, Cambridge, UK) or a Picro-Sirius Red solution (Abcam, Cambridge, UK) according to their manufacturer’s protocols. For Oil Red O staining, liver cryostat-sectioned slides were exposed to pure propylene glycol, then moved to 0.5% Oil Red O solution at 60 °C, followed by soaking in 85% propylene glycol. The slides were then rinsed and counterstained with hematoxylin, rinsed with water, and then mounted with aqueous mounting reagent. The slides were imaged using a Keyence BZ-x710 brightfield microscope (Keyence, Osaka, Honshu, Japan).

### 4.10. Fluorescence-Activated Cell Sorting

Cells were dispersed from the liver tissue and macrophages were isolated from dispersed cells using fluorescence-activated cell sorting (FACS) as previously described [48]. Macrophage cells were incubated with anti-mouse CD16/32 and PE anti-mouse CD64 followed by washing with PBS/1% FBS. Resuspended cells were incubated with Hoechst 33258 (1 µg/mL; Biotium, Fremont, CA, USA) added as a cell viability marker. Cells were sorted and analyzed using the BD FACSAria II (BD Biosciences, San Jose, CA, USA). BD FACSDiva 8.0 and TreeStar Flowjo (Ashland, OR, USA) were used to analyze data to generate flow cytometry images.

### 4.11. Statistical Analysis

The experimental data are shown as the mean ± SEM. Two-tailed unpaired Student’s *t*-test, area under the curve calculations, and one-way or two-way analysis of variance (ANOVA) were used for all mouse experiments using GraphPad Prism 9 software. For all tests, *p*-values less than 0.05 were considered statistically significant.

## Data Availability

The data supporting this study’s findings are available on request to the corresponding author.

## Supplementary Materials

The following supporting information can be downloaded at: https://www.mdpi.com/article/10.3390/ijms25042266/s1.
