# Supplementary material for: Sustained Effectiveness and Safety of Therapeutic miR-10a/b in Alleviating Diabetes and Gastrointestinal Dysmotility without Inducing Cancer or Inflammation in Murine Liver and Colon
Source: Int J Mol Sci. 2024 Feb 14;25(4):2266. doi: 10.3390/ijms25042266 (PMC10888952; doi:10.3390/ijms25042266)
Supplement: Supplementary file 1 [file ijms-25-02266-s001.zip › ijms-2829145-supplementary.pdf]

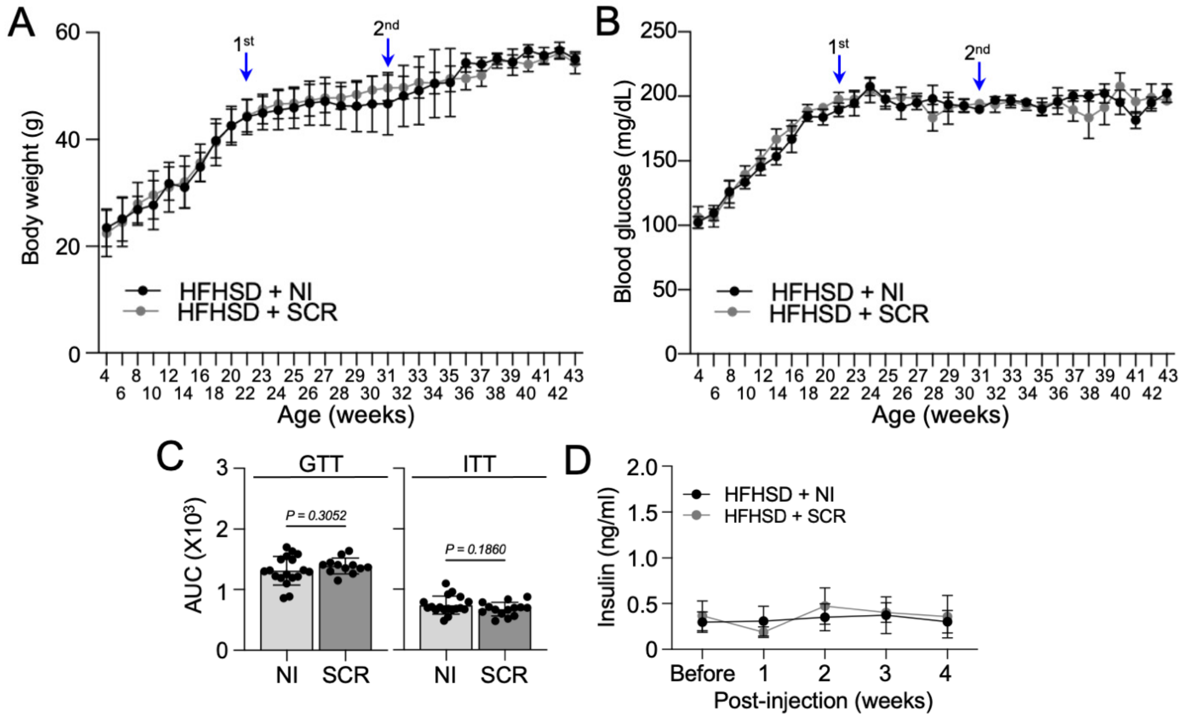

Supplementary Figure S1. No phenotypic changes observed in high-fat and high-sucrose diet (HFHSD) fed mice injected with a scramble RNA. C57BL/6 male mice injected twice at 22 and 31 weeks with a scramble RNA (SCR: 500 ng/g) or given no injection (NI). (A, B) Body weight and fasting blood glucose comparison. (C) Glucose and insulin tolerance test (GTT and ITT) plots of the area under the curve (AUC) comparison (D) Comparison of 6-hour fasting insulin levels in plasma. n=12-18 per condition for each experiment. Error bar indicates mean ± SEM.
